# Integrated Metabolomic and Transcriptomic Analysis Reveals the Pharmacological Effects and Differential Mechanisms of Isoflavone Biosynthesis in Four Species of *Glycyrrhiza*

**DOI:** 10.3390/ijms26062539

**Published:** 2025-03-12

**Authors:** Yuanfeng Lu, Zhen Ding, Daoyuan Zhang, Fuyuan Zhu, Bei Gao

**Affiliations:** 1 State Key Laboratory of Ecological Safety and Sustainable Development in Arid Lands, Xinjiang Institute of Ecology and Geography, Chinese Academy of Sciences, Urumqi 830011, China; luyuanfeng@njfu.edu.cn (Y.L.); dingzhen22@mails.ucas.ac.cn (Z.D.); zhangdy@ms.xjb.ac.cn (D.Z.); 2 Xinjiang Key Lab of Conservation and Utilization of Plant Gene Resources, Xinjiang Institute of Ecology and Geography, Chinese Academy of Sciences, Urumqi 830011, China; 3 The Southern Modern Forestry Collaborative Innovation Center, State Key Laboratory of Tree Genetics and Breeding, Key Laboratory of State Forestry and Grassland Administration on Subtropical Forest Biodiversity Conservation, College of Life Sciences, Nanjing Forestry University, Nanjing 210008, China; 4 University of Chinese Academy of Sciences, Beijing 100049, China

**Keywords:** licorice, metabolomics, transcriptomics, isoflavone biosynthesis, network pharmacology, potential distribution

## Abstract

Licorice (*Glycyrrhiza* L.) is a globally popular medicinal and edible plant, with nearly 30 species distributed across all continents. The usable part is primarily the root. To understand the metabolic differences among different *Glycyrrhiza* species, we selected four species and performed comprehensive analyses of their roots. Metabolomic profiling was conducted using UPLC-MS/MS and GC-MS, while transcriptomic analysis was carried out using RNA-sequencing. A total of 2716 metabolites were identified, including flavonoids (527 types) and terpenoids (251 types), among various other components. Subsequently, network pharmacology was employed to explore the medicinal value and potential pharmacological ingredients of these metabolites. Joint analysis of transcriptomic and metabolomic data revealed significant differences in differentially accumulated metabolites (DAMs) and differentially expressed genes (DEGs) in pairwise comparisons among the four species. These differences were primarily enriched in the isoflavone pathway. Further investigation into the regulatory mechanisms of isoflavone biosynthesis in different *Glycyrrhiza* species identified key genes and metabolites involved in isoflavone biosynthesis. Finally, we made reasonable predictions of the potential suitable habitats for the four *Glycyrrhiza* species, aiming to provide new insights for the development and utilization of licorice resources. The results of this study can serve as a basis for the development and utilization of licorice and for in-depth research on the regulation of isoflavone biosynthesis in licorice.

## 1. Introduction

Licorice is the common name for various plants of the genus *Glycyrrhiza* in the Fabaceae family. Members of this genus are distributed across Europe, Asia, South America, and North America, with the majority found in Eurasia [1]. Licorice has been widely used in both the food and medicinal fields from ancient times to the present. Its use dates back to 500 BC [2], with over 2000 years of documented use in Chinese medicine, first recorded in the “Shennong’s Classic of Material Medical” during the Han Dynasty [3]. Traditional Chinese medicine (TCM) regards licorice as a harmonizing agent that can mitigate the harsh properties or reduce the side effects of other drugs, earning it the title of “national elder” for its ability to harmonize various medications. Licorice is mentioned in all historical Chinese herbal texts, and the saying “nine out of ten prescriptions contain licorice” highlights its extensive clinical use [4]. Known as the “king of herbs”, modern pharmacology has confirmed that licorice possesses numerous pharmacological properties [5], including anti-inflammatory [6], antioxidant [7], antitumor [8], anti-ulcer, antiviral [9], antibacterial [10], and hepatoprotective [11] effects. The pharmacological activity of licorice primarily stems from its various active plant compounds, such as triterpenoid saponins [12], flavonoids [13], and other compounds, such as coumarins [14]. Flavonoids are plant-derived polyphenolic compounds with various bioactive functions, including antioxidative [15], anti-inflammatory [16], and cardiovascular protective effects [17]. Isoflavones, an important class of flavonoids mainly found in leguminous plants [18], play a crucial role in the pharmacological effects of licorice. Due to their molecular structure being similar to human estrogen, they are recognized as phytoestrogens and have positive effects on human health [19]. They also significantly contribute to the medicinal value of licorice, such as cardiovascular protection [20], neuroprotection [21], and anticancer effects [22]. Additionally, isoflavones act as signaling molecules in the symbiosis between rhizobia and legumes [23], playing an essential role in the growth, development, and environmental interactions of leguminous plants. The biosynthetic pathway of isoflavonoids in legumes is currently well investigated, comprising three key phases: the phenylpropanoid pathway, the biosynthesis of the isoflavonoid aglycone, and the final production of isoflavonoids [24]. An in-depth study of isoflavonoid biosynthesis not only helps reveal the chemical composition characteristics of licorice but also provides the scientific basis for the identification of its key active components, thus better elucidating its pharmacological mechanisms. Moreover, further research on isoflavonoid biosynthesis may enhance the resilience and ecological adaptability [25] of licorice plants [26], potentially paving the way for increased production of isoflavones, thereby aiding the production of functional foods [27]. The components of licorice have long been studied [13,28], and so far more than 400 compounds have been isolated from the genus *Glycyrrhiza* [29], with flavonoids and triterpenoid saponins being particularly abundant in the roots or rhizomes of licorice. However, current research on the chemical constituents of licorice mainly focuses on selected categories of compounds [30,31,32]. Like other natural functional foods and traditional herbs, the components of licorice are complex and not fully elucidated. Most existing studies rely solely on UPLC-MS analytical techniques [16,17,18], which, while powerful, provide limited coverage of the full metabolic landscape. Despite extensive research, a comprehensive metabolomic approach integrating multiple analytical techniques is still lacking, leaving the chemical diversity of licorice insufficiently explored. Additionally, nearly all current studies have focused on *Glycyrrhiza uralensis*, the most widely cultivated species, while other *Glycyrrhiza* species with potential medicinal or economic value have been largely overlooked. Addressing these gaps is crucial for a more complete understanding of the metabolic diversity and pharmacological potential of licorice species, which also aids in formulating strategies to combat drug resistance [33]. Simultaneously, the rapid growth of the licorice market has been accompanied by overharvesting and consequent ecological damage [34]. Licorice is an excellent vegetation for wind and sand control as well as for the improvement of saline-alkali land. Overharvesting of licorice can lead to increased soil desertification, more frequent sandstorms, and other catastrophic weather events [35]. To effectively meet the growing global demand, it is essential to establish sustainable and resilient global licorice production [36]. This involves curbing the trend of excessive wild licorice harvesting while actively developing and utilizing new licorice resources.

In this study, we selected four species of *Glycyrrhiza* plants: *Glycyrrhiza inflata* (Ginf), *Glycyrrhiza uralensis* (Gura), *Glycyrrhiza pallidiflora* (Gpal), and *Glycyrrhiza aspera* (Gasp). Among these, Ginf and Gura are listed in the “Chinese Pharmacopoeia [37]” and are traditional medicinal *Glycyrrhiza* species. Gpal is mainly distributed in eastern China and the Russian Far East, where its habitats are widespread yet sparsely populated. In agricultural practice, the stems and leaves of Gpal are commonly used as green manure, while its roots remain underutilized [38]. Gasp is widely distributed in Central Asia [39] and is present in small quantities in some regional licorice products [40]; however, its application has largely remained at the folk level without a scientifically established strategy for development. Therefore, Gpal and Gasp were included in this study as wild licorice resources with potential for development and utilization to validate their medicinal value or edibility.

This study utilized comprehensive metabolomic analyses using ultra-performance liquid chromatography–mass spectrometry (UPLC-MS) and gas chromatography–mass spectrometry (GC–MS) to extensively examine the differences and similarities in metabolites among the four *Glycyrrhiza* species: Ginf, Gura, Gpal, and Gasp. By integrating metabolomics and network pharmacology, potential pharmacological components of licorice for the treatment of cardiovascular system diseases were preliminarily screened. However, as metabolites serve as the final phenotypic manifestations of biological activities, even subtle changes can be exponentially amplified at the metabolic level, and standalone metabolite detection cannot elucidate the genetic regulatory mechanisms. To address the limitations of traditional single-omics studies, transcriptomic analysis was conducted, which further captured a wealth of differentially expressed genes and regulatory networks. The integration of metabolomics and transcriptomics provides an innovative strategy: metabolomics precisely identifies bioactive substances, while transcriptomics reveals the expression characteristics of pathway-related genes. Through this comprehensive approach, we systematically revealed the differences in isoflavone compound content and the expression of isoflavone biosynthesis-related genes among the different *Glycyrrhiza* species from both genetic regulatory and metabolic phenotypic perspectives. Finally, based on compiled species distribution data and environmental data, the MaxEnt model was used to predict the potential suitable habitats for the four *Glycyrrhiza* species under current climatic conditions. This study enhances our understanding of the active components and pharmacological effects of licorice and provides a theoretical basis for the further development and utilization of licorice resources.

## 2. Results and Discussion

### 2.1. Detection of Metabolites in the Roots of Four Glycyrrhiza Species

**Figure 1:**
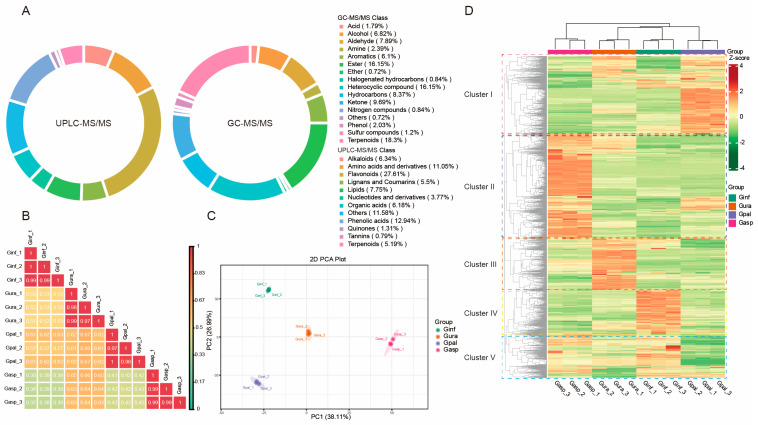
(**A**) Classification of 2716 metabolites detected by UPLC-MS/MS and GC-MS in the four types of licorice. (**B**) Correlation analysis. (**C**) PCA score plot. The sample groups are color-coded: red for *Glycyrrhiza aspera*; blue for *Glycyrrhiza pallidiflora*; green for *Glycyrrhiza inflata*; and orange for *Glycyrrhiza uralensis*. (**D**) Hierarchical clustering analysis (HCA) of secondary metabolites, with metabolites categorized into clusters. The normalized signal intensities of the metabolites are visualized as a color spectrum, where different colors represent different relative levels (red indicates high levels, green indicates low levels).

The health-promoting effects of licorice herbs are primarily attributed to their metabolites. To comprehensively understand the differences in metabolites in the roots of the four *Glycyrrhiza* species, this study analyzed root samples of Ginf (*Glycyrrhiza inflata*), Gura (*Glycyrrhiza uralensis*), Gpal (*Glycyrrhiza pallidiflora*), and Gasp (*Glycyrrhiza aspera*) using a dual detection platform of UPLC-MS/MS and GC-MS, along with a self-constructed database (Metware Database, MWDB). The good repeatability and reliability of UPLC-MS/MS and GC-MS analyses were confirmed by the high overlapping total ion current (TIC) chromatograms of the QC sample (Supplementary Figure S1). As a result, a total of 2716 metabolites were detected (Supplementary Table S1). Using the UPLC-MS/MS detection platform, a total of 1909 metabolites were identified. These compounds can be categorized into 12 classes: 527 flavonoids (27.61%), 247 phenolic acids (12.94%), 211 amino acids and derivatives (11.05%), 148 lipids (7.75%), 121 alkaloids (6.34%), 118 organic acids (6.18%), 105 lignans and coumarins (5.5%), 99 terpenoids (5.19%), 72 nucleotides and derivatives (3.77%), 25 quinones (1.31%), 15 tannins (0.79%), and 221 other metabolites (11.58%) (Figure 1A). Among these, 1866 and 1887 metabolites were identified in Ginf and Gura, respectively, which are recognized as medicinal *Glycyrrhiza* species. In comparison, 1886 and 1870 metabolites were identified in Gpal and Gasp, respectively. Overall, 1797 metabolites were found to be common across all four *Glycyrrhiza* species.

Using the GC-MS detection platform, a total of 836 metabolites were identified. These compounds can be divided into 16 categories: 135 esters (16.15%), 153 terpenoids (18.3%), 135 heterocyclic compounds (16.15%), 70 hydrocarbons (8.37%), 57 alcohols (6.82%), 81 ketones (9.69%), 66 aldehydes (7.89%), 51 aromatics (6.1%), 17 phenols (2.03%), 15 acids (1.79%), 7 nitrogen compounds (0.84%), 20 amines (2.39%), 7 halogenated hydrocarbons (0.84%), 10 sulfur compounds (1.2%), 6 ethers (0.72%), and 6 others (0.72%) (Figure 1A). Among these, 751, 706, 821, and 700 volatile metabolites were detected in Ginf, Gura, Gpal, and Gasp samples, respectively, with 622 volatile metabolites being common to all four *Glycyrrhiza* species.

In previous LC-MS-based studies on licorice metabolites conducted in the past three years, the number of metabolites reported was 996 [41], 866 [42], and 831 [43], respectively. In contrast, our LC + GC full-spectrum metabolomic approach detected a total of 2745 metabolites (1909 from UPLC-MS/MS plus 836 from GC-MS), representing an approximately 2.8- to 3.3-fold increase in the number of identified metabolites compared to previous studies. This substantial increase in metabolite coverage demonstrates that the LC + GC-based full-spectrum metabolomics method is a powerful and effective tool for comprehensive plant metabolite identification. Its enhanced detection capability significantly improves the coverage and accuracy of metabolite analysis, thereby providing a solid technical foundation for subsequent in-depth studies of the chemical components and biological activities of licorice. Correlation analysis of metabolites among different licorice samples (Figure 1B) showed that Gura and Gpal (0.61 ≤ |r| ≤ 0.67), as well as Gura and Gasp (0.63 ≤ |r| ≤ 0.66), exhibited relatively similar distribution patterns, indicating a strong correlation between the metabolites of these Glycyrrhiza species. In contrast, Gasp and Ginf (0.37 ≤ |r| ≤ 0.39) and Gasp and Gpal (0.41 ≤ |r| ≤ 0.42) exhibited weaker correlations. Interestingly, the two Glycyrrhiza species long used as medicinal herbs, Ginf and Gura, showed a high correlation only in the GC-MS assessment (0.93 ≤ |r| ≤ 0.97). Unexpectedly, Gasp also demonstrated a strong correlation with both Ginf (0.89 ≤ |r| ≤ 0.94) and Gura (0.92 ≤ |r| ≤ 0.95) in the GC-MS assessment (Supplementary Figure S2). This suggests that Gasp has a similar flavor profile to the traditional medicinal Glycyrrhiza species (Ginf and Gura).

Among all metabolites in the four Glycyrrhiza species, flavonoids (527 compounds) are the most abundant. The flavonoids in licorice mainly include flavones (145 compounds), flavonols (108 compounds), chalcones (37 compounds), flavanones (61 compounds), isoflavones (86 compounds), and chalcones (37 compounds). Several of these compounds have proven pharmacological properties. For example, isoliquiritigenin has been demonstrated to have antioxidative and neuroprotective mechanisms [44], as well as significant antitumor effects [45]. Glabridin exhibits multiple actions, including anti-inflammatory, antioxidative, antitumor, antimicrobial, bone protective, cardiovascular protective, neuroprotective, hepatoprotective, anti-obesity, and antidiabetic effects [46]. Other compounds, such as liquiritin [47], glycyrrhizic acid, and glycyrrhetinic acid [48], have received extensive attention and have been comprehensively studied. Next in abundance are terpenoids (251 compounds), which are widely recognized for their antitumor [49], antihyperglycemic, and antidiabetic effects [50]. The terpenoids in licorice primarily include isoglycyrrhizic acid, hypoglycyrrhizic acid (β), and glycyrrhizic acid, with glycyrrhizic acid being a particularly important component. It plays significant roles in antiviral [51], anti-inflammatory [52], antitumor [53], and heart failure treatments [54]. Additionally, due to its unique sweetness, which is over 50 times that of sucrose, glycyrrhizic acid is an important sweetener [55]. It also serves as a good surfactant, used as a foaming agent in products like beverages, candies, and sweets to improve their foaming properties. In fermented beverages such as beer, it can stabilize and refine the foam, reducing any potential aftertaste bitterness [28,56]. Other metabolites with relatively high abundance include phenolic acids and amino acids and derivatives.

### 2.2. Multivariate Statistical Analysis

To determine the metabolic differences among the four *Glycyrrhiza* species, PCA and HCA analyses were first conducted. The PCA results showed that biological replicates of the four *Glycyrrhiza* species clustered together in different regions, while there was a clear separation between the species (Figure 1C). The two principal components (PC1 and PC2) contributed to 65.10% of the cumulative variance, with PC1 and PC2 accounting for 38.11% and 26.99% of the variance, respectively. Notably, PC1 effectively separated Gasp from Ginf, Gpal, and Gura, while PC2 separated Ginf and Gpal from Gura and Gasp. This indicates significant differences between the *Glycyrrhiza* species, with each species exhibiting good reproducibility.

Additionally, HCA was performed based on the relative abundance of metabolites (Figure 1D). The HCA heatmap clearly showed that metabolites could be effectively divided into five clusters. The distribution of these secondary metabolites in each cluster was as follows: Cluster i had the highest levels in Gpal, Cluster ii in Gasp, Cluster iii in Gura, Cluster iv in Ginf, and Cluster V had high levels in both Gasp and Ginf. Consistent with the PCA results, the HCA analysis also indicated significant differences in metabolites among the different *Glycyrrhiza* species.

OPLS-DA was used to further screen the differential metabolites between the pairwise comparisons of the four *Glycyrrhiza* species. According to the OPLS-DA score plots (Supplementary Figure S3A–F), significant separation was observed between the different *Glycyrrhiza* species. The evaluation metrics R2Y and Q2 in the OPLS-DA models for the comparisons were all greater than 0.9, indicating that the model had a high fitting accuracy. These results confirm that each of the four *Glycyrrhiza* species possesses distinct metabolic profiles.

### 2.3. Differential Analysis of Metabolites in Different Licorice Roots

**Figure 2:**
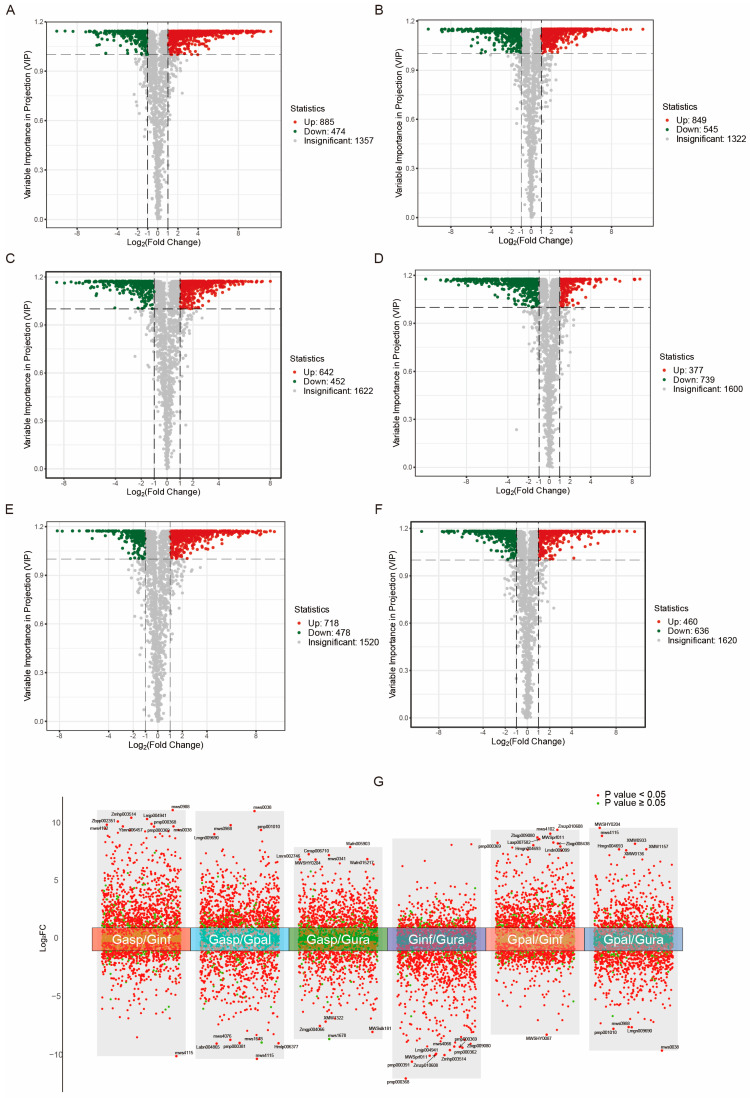
Volcano plots depicting the differential metabolite expression levels across various licorice comparison groups. (**A**) *Glycyrrhiza aspera* vs. *Glycyrrhiza inflata*. (**B**) *Glycyrrhiza aspera* vs. *Glycyrrhiza pallidiflora*. (**C**) *Glycyrrhiza aspera* vs. *Glycyrrhiza uralensis*. (**D**) *Glycyrrhiza inflata* vs. *Glycyrrhiza uralensis*. (**E**) *Glycyrrhiza pallidiflora* vs. *Glycyrrhiza inflata*. (**F**) *Glycyrrhiza pallidiflora* vs. *Glycyrrhiza uralensis*. (**G**) Overall multi-group differential volcano plot for all comparison groups, showcasing the top 10 most significant differential metabolites in each comparison.

Differential metabolites were screened using VIP ≥ 1 and fold change ≥ 2 or ≤0.5 and displayed using volcano plots (Figure 2A–F). There were 1359, 1394, 1094, 1116, 1196, and 1096 differential metabolites in the comparisons of Gasp vs. Ginf, Gasp vs. Gpal, Gasp vs. Gura, Ginf vs. Gura, Gpal vs. Ginf, and Gpal vs. Gura, respectively. These results strongly suggest significant differences in the composition and regulation of metabolites among different *Glycyrrhiza* species. Notably, most of the differential metabolites belonged to categories such as flavonoids, phenolic acids, amino acids, and derivatives (Supplementary Table S2), which are known to have various bioactive properties and are considered to be related to the main medicinal value of licorice [57,58,59].

**Figure 3:**
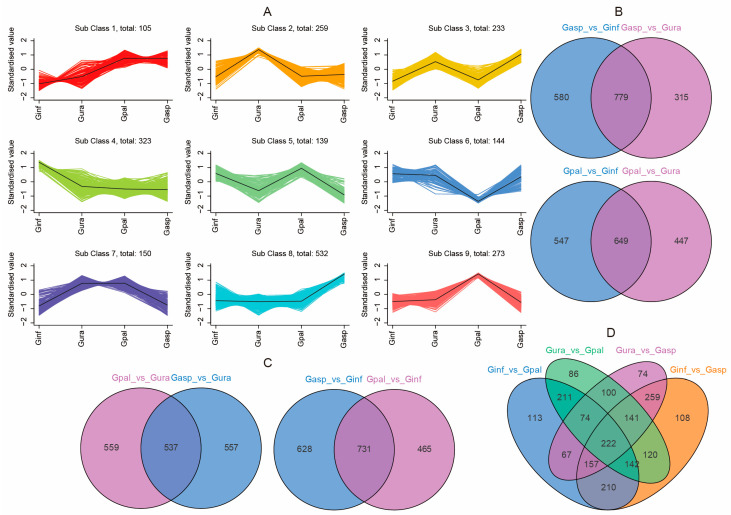
Differential metabolomic analysis of the four types of licorice. (**A**) K-means plot of differential metabolites. The x-axis represents the samples, and the y-axis represents the relative abundance of standardized metabolites. (**B**,**C**) Venn diagrams of differential metabolites for each group comparison: (**B**) *Glycyrrhiza aspera* vs. *Glycyrrhiza inflata*/*Glycyrrhiza aspera* vs. *Glycyrrhiza uralensis*, *Glycyrrhiza pallidiflora* vs. *Glycyrrhiza inflata*/*Glycyrrhiza pallidiflora* vs. *Glycyrrhiza uralensis*. (**C**) *Glycyrrhiza pallidiflora* vs. *Glycyrrhiza uralensis*/*Glycyrrhiza aspera* vs. *Glycyrrhiza uralensis*, *Glycyrrhiza aspera* vs. *Glycyrrhiza inflata*/*Glycyrrhiza pallidiflora* vs. *Glycyrrhiza inflata*. (**D**) Venn diagram of differential metabolites in multiple pairwise comparisons: *Glycyrrhiza inflata* vs. *Glycyrrhiza pallidiflora*/*Glycyrrhiza uralensis* vs. *Glycyrrhiza pallidiflora*/*Glycyrrhiza uralensis* vs. *Glycyrrhiza aspera*/*Glycyrrhiza inflata* vs. *Glycyrrhiza aspera*. Different colors represent different sets, overlapping areas represent the intersections of different comparison groups, and the numbers indicate the number of shared metabolites. The non-overlapping areas indicate the number of unique metabolites in each comparison group.

Additionally, to study the relative abundance trends of metabolites in different groups, a k-means plot (Figure 3A) was used to cluster the relative abundances of all screened differential metabolites. The relative abundance of metabolites in the four Glycyrrhiza species showed significant differences, with all differential metabolites divided into nine sub-classes. Among them, sub-class 2, sub-class 4, sub-class 8, and sub-class 9 contained 259, 323, 532, and 273 differential metabolites, respectively, which had higher abundances in Gura, Ginf, Gasp, and Gpal. This indicates that the metabolite content in Gasp differs considerably from the other three Glycyrrhiza species. In sub-class 1, the metabolite content in Gasp and Gpal was higher than in Ginf and Gura. In sub-class 3, the metabolite content in Gura and Gasp was higher than in Ginf and Gpal, while sub-class 5 exhibited the opposite trend. In sub-class 7, the metabolite content in Gura and Gpal was higher than in Ginf and Gasp.

To further understand the differences in metabolites among the various *Glycyrrhiza* species, pairwise comparisons were conducted, and the relationships between the differential metabolites were displayed using Venn diagrams. It was found that there were 779 differential metabolites common to Gasp compared to both Gura and Ginf and 649 differential metabolites common to Gpal compared to both Gura and Ginf (Figure 3B). For Gasp and Gpal, two wild *Glycyrrhiza* species with potential but underutilized value, there were 537 differential metabolites common to both compared to Gura and 731 compared to Ginf (Figure 3C). Finally, an overall analysis of the differential metabolites among the different *Glycyrrhiza* species showed that Gasp and Gpal had 222 differential metabolites common compared to both Gura and Ginf (Figure 3D).

### 2.4. Network Pharmacology of Licorice

#### 2.4.1. Major Medicinal Components of Licorice

Licorice has been highly valued in traditional Chinese medicine (TCM) and is widely used in prescriptions. Its medicinal properties were documented over 2400 years ago in European and Middle Eastern texts [2]. Research on licorice has never ceased, revealing numerous active ingredients and their mechanisms over the past few decades [60,61]. However, the comprehensive detection of licorice components based on extensively targeted metabolites is more thorough than previous identifications.

**Figure 4:**
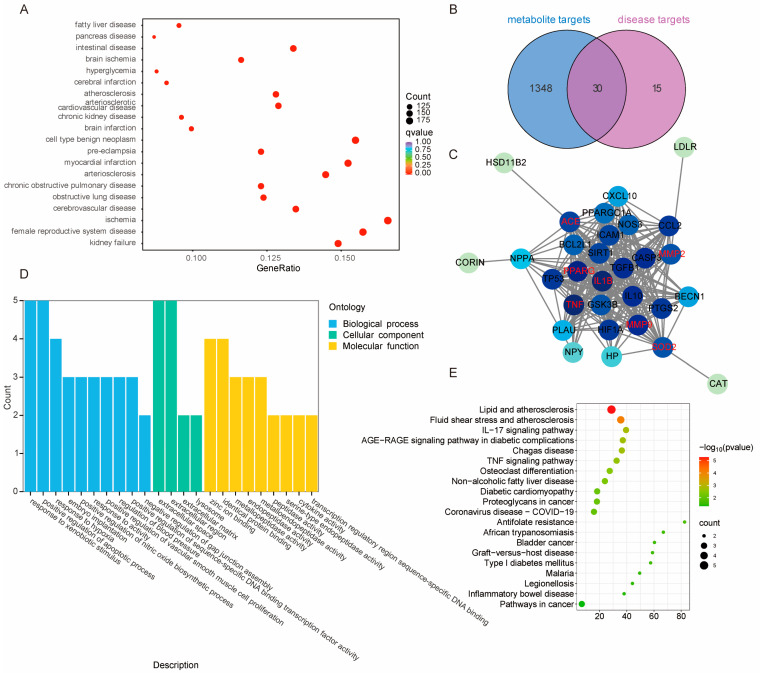
Network pharmacology analysis of four licorice species. (**A**) Enrichment analysis of the top 20 Disease Ontology (DO) terms with *p*-value < 0.05. (**B**) Venn diagram showing the overlap of licorice targets with cardiovascular system disease. (**C**) PPI network construction diagram of overlapping targets, with red nodes indicating core targets selected. (**D**) Gene Ontology (GO) entries related to Biological Processes (BP), Cellular Components (CC), and Molecular Functions (MF), with the top 10 terms displayed for each category if more than 10 entries are available. (**E**) Enrichment analysis of the top 20 KEGG pathways associated with core targets.

Therefore, we further screened all detected substances for active ingredients using three databases—TCMSP, SwissDrugDesign, and SymMap—to determine the key active components promoting human health in the four *Glycyrrhiza* species. Our comprehensive analysis identified 299 metabolites as potential active ingredients in TCM, corresponding to 2044 substance targets. These targets were then subjected to Disease Ontology (DO) enrichment analysis to link the targets with diseases (Supplementary Table S3). Our disease enrichment analysis revealed that the substance targets are related to multiple diseases. We screened the top 20 diseases with a *p*-value < 0.05 (Figure 4A) and found that the majority belonged to cardiovascular system diseases (six diseases). The next most common were respiratory system diseases (three diseases) and urinary system diseases (two diseases). Additionally, five diseases belonged to multiple major categories, with four of these falling under both cardiovascular and nervous system diseases. Therefore, we believe that licorice can effectively target cardiovascular system diseases, respiratory system diseases, urinary system diseases, and nervous system diseases.

We further conducted a network pharmacology analysis on the top 10 diseases within the cardiovascular system disease category. We retrieved 45 targets related to these diseases from the DisGeNET, SymMap, and TTD databases. Further Venn diagram analysis identified 30 common targets between licorice active ingredients and these diseases (Figure 4B). These common targets were added to the STRING 20.0 database. To further identify licorice’s effects on cardiovascular system diseases, we used Cytoscape 3.10.1 software to screen core targets, selecting targets with degree, closeness, and betweenness values greater than the average as core targets. Finally, we identified seven core targets for licorice and cardiovascular system diseases (Figure 4C).

#### 2.4.2. Gene Ontology (GO) and Kyoto Encyclopedia of Genes and Genomes (KEGG) Enrichment Analysis

To further understand the mechanisms by which licorice treats various cardiovascular system diseases, we performed GO functional and KEGG pathway enrichment analysis on the seven core target genes using DAVID. The analysis results showed that 82, 4, and 9 GO entries related to biological process (BP), cellular component (CC), and molecular function (MF). We presented the top 10 entries of Biological Processes (BP) and the entries for the other two categories (Figure 4D). BP mainly includes response to xenobiotic stimulus, positive regulation of apoptotic process, response to hypoxia, etc.; CC entries mainly comprise extracellular space and extracellular regions, while MF enrichment involves zinc ion binding and identical protein binding.

Additionally, our analysis revealed a total of 36 signaling pathways associated with the core target genes, showing the top 20 signaling pathways (Figure 4E). It is noteworthy that the core target genes related to cardiovascular system disease mainly involve pathways such as lipid and atherosclerosis, fluid shear stress and atherosclerosis, IL-17 signaling pathway, AGE-RAGE signaling pathway in diabetic complications, etc. Importantly, most of the enriched pathways are related to anti-inflammatory processes, suggesting that licorice exerts its therapeutic effect on cardiovascular system disease by influencing inflammation-related pathways [38,39]. These findings indicate that modulating these inflammation-related pathways may serve as a potential strategy for treating cardiovascular system diseases. Future research could further explore the specific roles and prospects of these pathways in the treatment of cardiovascular diseases [62,63]. These results suggest that licorice exerts its therapeutic effects on cardiovascular system diseases by modulating inflammation-related pathways. Consequently, targeting these inflammation-related pathways might be a potential strategy for treating cardiovascular system diseases. Future research could further explore the specific roles and therapeutic potential of these pathways in the treatment of cardiovascular diseases.

### 2.5. Transcriptome Analysis of Four Glycyrrhiza Species

**Figure 5:**
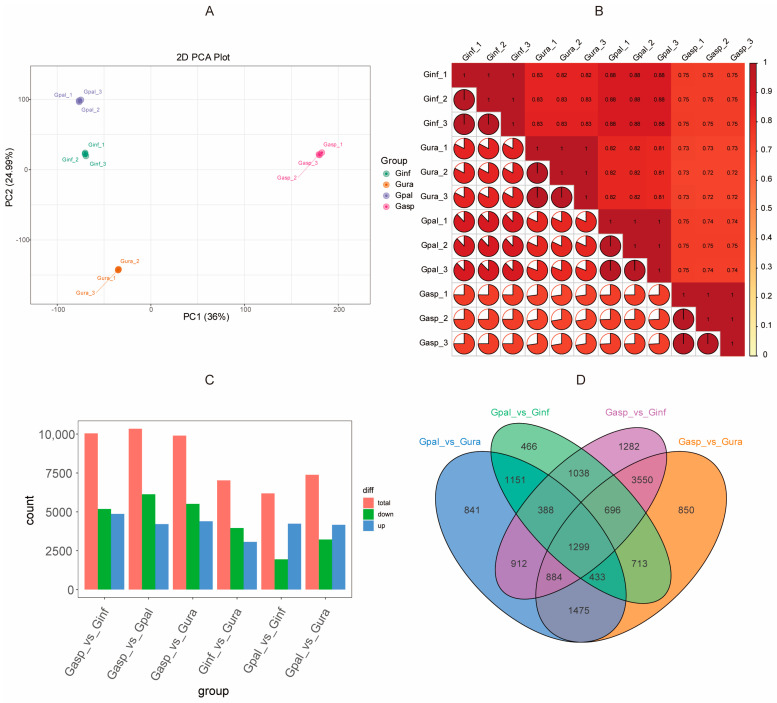
Transcriptome analysis of four licorice species. (**A**) PCA score plot. Sample groups are color-coded as follows: red, *Glycyrrhiza aspera*; blue, *Glycyrrhiza pallidiflora*; green, *Glycyrrhiza inflata*; orange, *Glycyrrhiza uralensis*. (**B**) Correlation analysis. (**C**) Overall characteristics bar plot of differentially expressed genes (DEGs) in each comparison group. (**D**) Venn diagram of DEGs in multiple pairwise comparisons (*Glycyrrhiza pallidiflora* vs. *Glycyrrhiza uralensis*/*Glycyrrhiza pallidiflora* vs. *Glycyrrhiza inflata*/*Glycyrrhiza aspera* vs. *Glycyrrhiza inflata*/*Glycyrrhiza aspera* vs. *Glycyrrhiza uralensis*). Different colors represent different sets, overlapping areas represent intersections of different comparison groups, and numbers indicate the number of shared DEGs. Numbers in non-overlapping areas represent the number of unique DEGs in each comparison group.

Transcriptome sequencing was performed on the roots of four *Glycyrrhiza* species. After filtering raw data and checking sequencing error rates and GC content distribution, we obtained 82.95 Gb of clean data from 12 samples, with each sample yielding over 6 Gb of clean data and a Q30 base percentage of 94% or higher. Post quality control, the clean reads were aligned to the *Glycyrrhiza uralensis* reference genome (Bioproject ID: PRJDB3943) [64], with alignment rates ranging from 79.90% to 94.92% and an average alignment rate of 89.99% (Table 1). This indicates that the high-quality RNA-seq data is suitable for further analysis. Principal Component Analysis (PCA) (Figure 5A) and a correlation heatmap (Figure 5B) confirmed the high consistency among samples within the same group, making them suitable for Differentially Expressed Genes (DEG) analysis. To comprehensively understand the functions of the genes obtained from transcriptome analysis, gene function annotation was performed using seven major databases (KEGG, NR, Swiss-Prot, Tremble, KOG, GO, and Pfam). Specifically, 19,561 genes were annotated based on the KEGG database; 13,350 genes based on the NR database; 20,353 genes based on the Swiss-Prot database; 11,558 genes based on the Tremble database; 15,178 genes based on the KOG database; 24,713 genes based on the GO database; and 23,833 genes based on the Pfam database (Supplementary Table S4).

### 2.6. Transcriptome Differences Among Four Glycyrrhiza Species

DESeq2 was used to identify differentially expressed genes (DEGs), with selection criteria of |log2 (fold change)| > 1 and a false discovery rate (FDR) < 0.05. The results showed 10,049, 10,341, 9900, 7021, 6184, and 7383 DEGs in the comparison groups of Gasp vs. Ginf, Gasp vs. Gpal, Gasp vs. Gura, Ginf vs. Gura, Gpal vs. Ginf, and Gpal vs. Gura, respectively. Further analysis focused on four main comparisons, revealing 4238 upregulated and 1946 downregulated genes in Ginf vs. Gpal; 3219 upregulated and 4164 downregulated genes in Gura vs. Gpal; 5508 upregulated and 4392 downregulated genes in Gura vs. Gasp; and 5183 upregulated and 4866 downregulated genes in Ginf vs. Gasp. The overall characteristics of DEGs in each comparison group are presented in bar charts (Figure 5C). Finally, an overall analysis of the differential metabolites among the four *Glycyrrhiza* species revealed that Gasp and Gpal, compared to Gura and Ginf, shared 1299 DEGs (Figure 5D).

### 2.7. Functional and Enrichment Analysis of Differential Metabolites and Differentially Expressed Genes

**Figure 6:**
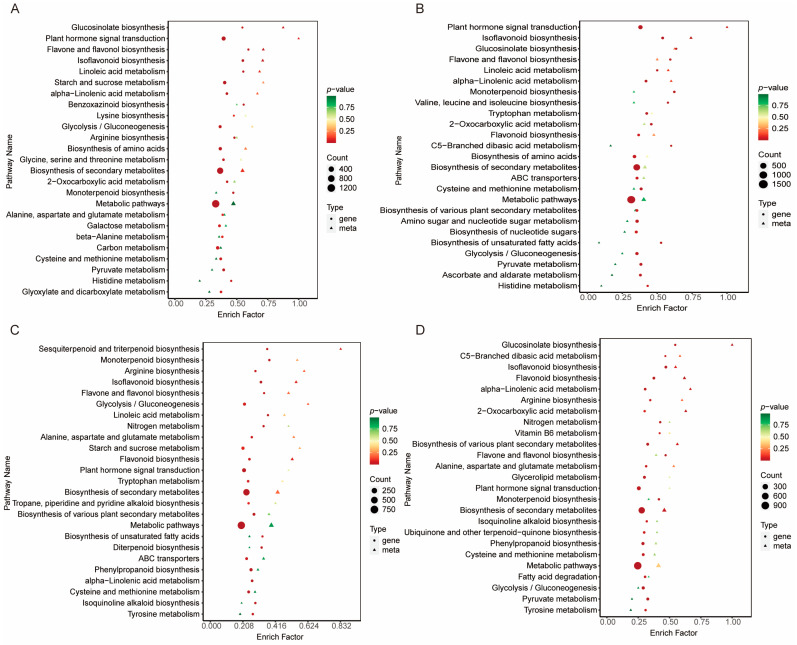
Distribution of KEGG annotation and enriched pathways of DEGs and DEMs in four comparison groups of licorice species. (**A**) *Glycyrrhiza aspera* vs. *Glycyrrhiza inflata*. (**B**) *Glycyrrhiza aspera* vs. *Glycyrrhiza uralensis*. (**C**) *Glycyrrhiza pallidiflora* vs. *Glycyrrhiza inflata*. (**D**) *Glycyrrhiza pallidiflora* vs. *Glycyrrhiza uralensis*.

A KEGG enrichment analysis was performed on the shared differential metabolites and differentially expressed genes (DEGs) across the various comparison groups. A combined analysis of 222 differential metabolites and 1299 DEGs from the four comparison groups was conducted. For each comparison, the top 25 most significantly enriched pathways were displayed in a scatter plot (Figure 6A–D). It was observed that in the Ginf vs. Gasp group, the differential metabolites and DEGs were primarily enriched in pathways such as glucosinolate biosynthesis, plant hormone signal transduction, flavone and flavonol biosynthesis, isoflavonoid biosynthesis, and linoleic acid metabolism. In the Gasp vs. Gura group, they were mainly enriched in plant hormone signal transduction, isoflavonoid biosynthesis, glucosinolate biosynthesis, flavone and flavonol biosynthesis, and linoleic acid metabolism. In the Gpal vs. Ginf group, the main enrichments were in sesquiterpenoid and triterpenoid biosynthesis, monoterpenoid biosynthesis, arginine biosynthesis, isoflavonoid biosynthesis, and flavone and flavonol biosynthesis. For the Gpal vs. Gura group, the differential metabolites and DEGs were primarily enriched in glucosinolate biosynthesis, C5-branched dibasic acid metabolism, isoflavonoid biosynthesis, flavonoid biosynthesis, and alpha-linolenic acid metabolism.

Some metabolic pathways were found to be overlapping across these comparison groups. For example, glucosinolate biosynthesis appeared three times among the top 10 most significant pathways in the four comparison groups, ranking first in significance in both the Gasp vs. Ginf and Gpal vs. Gura groups. Additionally, flavone and flavonol biosynthesis, along with two fatty acid metabolism pathways (linoleic acid metabolism and alpha-linolenic acid metabolism), appeared three times each in the top 10 KEGG pathways across the four groups. Notably, isoflavonoid biosynthesis was present in the top five for all four comparison groups, indicating significant differences in this pathway among the four *Glycyrrhiza* species.

### 2.8. Integrative Transcriptomics and Metabolomics Analysis Reveals Isoflavonoid Biosynthesis Mechanisms in Different Glycyrrhiza Species

The aforementioned KEGG enrichment analysis of differential metabolites and differentially expressed genes (DEGs) indicates that the primary differences among the four *Glycyrrhiza* species are concentrated in isoflavonoid biosynthesis. To understand the differences in isoflavonoid synthesis among the four species, we integrated transcriptomics and metabolomics data. Based on transcriptomic data, we identified 99 genes related to isoflavonoid biosynthesis. Metabolomic data revealed 27 metabolites. Through joint analysis, we further identified DEGs and DEMs that were significantly enriched and strongly correlated in the isoflavonoid biosynthesis pathway. Additionally, the correlations between DEGs and DEMs were determined with a Pearson correlation coefficient absolute value greater than 0.8 and a *p*-value less than 0.05.

**Figure 7:**
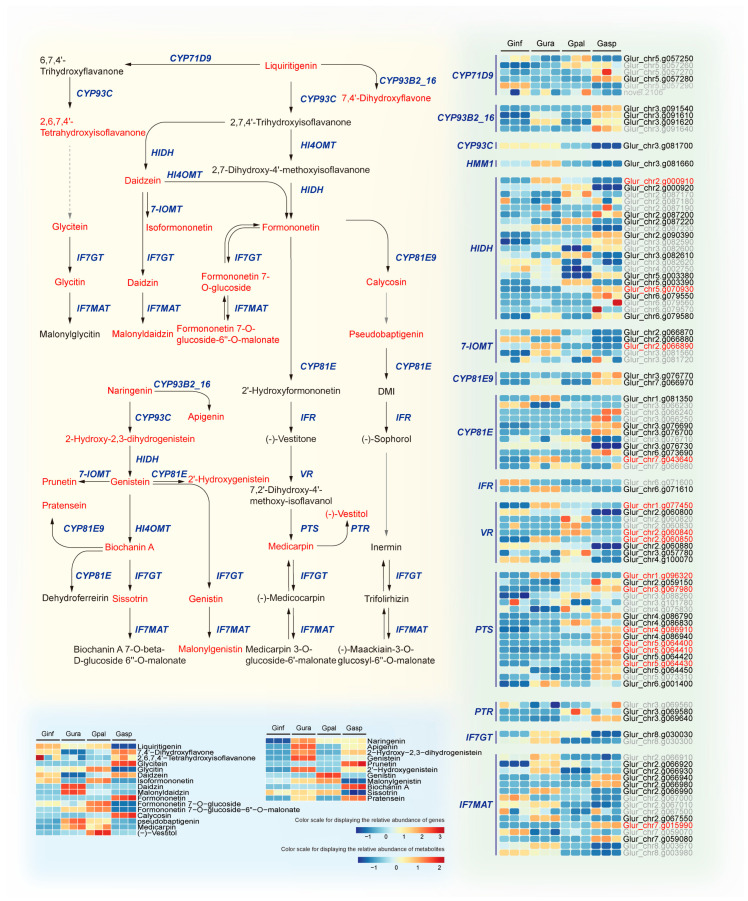
Genes and metabolites in the isoflavonoid biosynthesis pathway of four licorice species. In the pathway diagram, blue represents genes, while red and black represent metabolites, with red indicating metabolites detected in this study. In the expression levels and annotation information, genes and metabolites shown in black or red represent DEGs or DEMs, with red DEGs indicating significant differences across all four comparison groups. Gene expression levels between the different control groups are expressed as FPKM values, and metabolite levels are expressed as log2FC values.

We found that a total of 61 DEGs encode 14 key enzymes involved in isoflavonoid biosynthesis, including two flavonoid 6-hydroxylases (CYP71D9 [EC:1.14.13.-]), three flavone synthase IIs (CYP93B16 [EC:1.14.19.76]), one 2-hydroxyisoflavanone synthase (CYP93C [EC:1.14.14.87]), one 2,7,4′-trihydroxyisoflavanone 4′-O-methyltransferase (HMM1 [EC:2.1.1.212 2.1.1.46]), 11 2-hydroxyisoflavanone dehydratases (HIDH [EC:4.2.1.105]), three isoflavone-7-O-methyltransferases (7-IOMT [EC:2.1.1.150]), two isoflavone 3′-hydroxylases (CYP81E9 [EC:1.14.14.88]), six isoflavone 2′-hydroxylases (CYP81E [EC:1.14.14.90 1.14.14.89]), one 2′-hydroxyisoflavone reductase (IFR [EC:1.3.1.45]), seven vestitone reductases (VR [EC:1.1.1.348]), 13 pterocarpan synthases (PTS [EC:4.2.1.139]), two pterocarpan reductases (PTR [EC:1.23.1.-]), one isoflavone 7-O-glucosyltransferase (IF7GT [EC:2.4.1.170]), and eight isoflavone 7-O-glucoside-6″-O-malonyltransferases (IF7MAT [EC:2.3.1.115]). Notably, three genes, 7-IOMT (EC:2.1.1.150) (Glur_chr2.g066890), CYP81E (EC:1.14.14.90 1.14.14.89) (Glur_chr7.g043640), and IF7MAT (EC:2.3.1.115) (Glur_chr7.g015990), showed significant differences in all pairwise comparisons among the four *Glycyrrhiza* species. Additionally, HIDH (EC:4.2.1.105) (Glur_chr2.g000910, Glur_chr5.g070930), VR (EC:1.1.1.348) (Glur_chr1.g077450, Glur_chr2.g060840, Glur_chr2.g060850), and PTS (EC:4.2.1.139) (Glur_chr1.g096320, Glur_chr3.g067980, Glur_chr4.g086910, Glur_chr5.g064400, Glur_chr5.g064410, Glur_chr5.g064430) were also significantly different in all pairwise comparisons. Furthermore, 26 DEMs were detected. A comprehensive analysis of isoflavonoid biosynthesis in the four *Glycyrrhiza* species revealed the biosynthetic mechanisms, clearly showing the differences in isoflavonoid biosynthesis (Figure 7).

The correlation analysis indicated that CYP81E (EC:1.14.14.90 1.14.14.89) (Glur_chr1.g081350) and IFR (EC:1.3.1.45) (Glur_chr6.g071610) were highly correlated with Daidzin (PPC > 0.99), with both genes being highly expressed in Gura. PTS (EC:4.2.1.139) (Glur_chr1.g096320) and IF7MAT (EC:2.3.1.115) (Glur_chr2.g066990) were highly correlated with Malonyldaidzin (PPC > 0.99), with both genes being highly expressed in Gura. IF7MAT (EC:2.3.1.115) (Glur_chr2.g066980) and PTS (EC:4.2.1.139) (Glur_chr4.g086940, Glur_chr5.g064420) were highly correlated with Formononetin and Prunetin (PPC > 0.99). Additionally, PTS (EC:4.2.1.139) (Glur_chr4.g086940, Glur_chr5.g064420) was also highly correlated with Biochanin A (PPC > 0.99). PTS (EC:4.2.1.139) (Glur_chr5.g064420) was highly correlated with Glycitein and Calycosin (PPC > 0.99), and IF7MAT (EC:2.3.1.115) (Glur_chr7.g015990) was also highly correlated with Glycitein and Calycosin (PPC > 0.99). PTS (EC:4.2.1.139) (Glur_chr5.g064400) was highly correlated with Glycitein (PPC > 0.99). All these genes were highly expressed in Gsap, and the levels of these metabolites were higher in Gsap compared to the other three species. CYP81E9 (EC:1.14.14.88) (Glur_chr7.g066970) was highly correlated with Pratensein, and PTS (EC:4.2.1.139) (Glur_chr4.g086790) was highly correlated with Biochanin A (PPC > 0.99). Both genes were highly expressed in Gsap, with Pratensein and Biochanin A levels being higher in Gsap than in the other three species. HIDH (EC:4.2.1.105) (Glur_chr2.g000920) was highly correlated with Glycitin, and VR (EC:1.1.1.348) (Glur_chr2.g060840) was highly correlated with Genistin (PPC > 0.99). Both genes were highly expressed in Gpal, with Glycitin and Genistin levels being higher in Gpal than in the other three species. CYP93B16 (EC:1.14.19.76) (Glur_chr3.g091620) was highly correlated with 2-Hydroxy-2,3-dihydrogenistein (PPC > 0.99), and Glur_chr3.g091620 was highly expressed in Gura and Gsap, with 2-Hydroxy-2,3-dihydrogenistein levels also being relatively higher in Gura and Gsap. Apart from that, HIDH [EC:4.2.1.105] (Glur_chr2.g000910) shows a strong negative correlation (|PPC| > 0.99) with Daidzein. HIDH [EC:4.2.1.105] is highly expressed in Gura, whereas Daidzein content in Gura is lower compared to the other three licorice species. Similarly, CYP81E [EC:1.14.14.90 1.14.14.89] (Glur_chr2.g000910) exhibits a strong negative correlation (|PPC| > 0.99) with Formononetin. CYP81E [EC:1.14.14.90 1.14.14.89] is expressed at lower levels in Gasp, while Formononetin content in Gasp is higher compared to the other three licorice species. Additionally, PTS [EC:4.2.1.139] (Glur_chr5.g064430) shows a strong negative correlation (|PPC| > 0.99) with Liquiritigenin. PTS [EC:4.2.1.139] is highly expressed in Gasp, whereas Liquiritigenin content in Ginf, Gura, and Gpal is higher than in Gasp. These are key factors contributing to the differences in isoflavonoid biosynthesis among the different *Glycyrrhiza* species.

We conducted orthogonal partial least squares (O2PLS) analysis to identify key genes and metabolites associated with both transcriptomic and metabolomic profiles in licorice. To determine which metabolites and genes are interrelated, we depicted two omics correlation maps for the top 5 differentially expressed metabolites (DEMs) and the top 10 differentially expressed genes (DEGs) (Supplementary Figure S4). The analysis revealed that the most highly correlated DEMs were Genistein, Apigenin, Daidzin, 2′-Hydroxygenistein, and Malonyldaidzin. The top 10 DEGs comprised two CYP81E [EC:1.14.14.90], two PTS [EC:4.2.1.139], two VR [EC:1.1.1.348], and one each of IFR [EC:1.3.1.45], CYP93B2_16 [EC:1.14.19.76], IF7MAT [EC:2.3.1.115], and HIDH [EC:4.2.1.105]. These findings identify candidate hub genes and metabolites involved in isoflavone biosynthesis across different *Glycyrrhiza* species.

### 2.9. Potential Distribution Analysis of Four Glycyrrhiza Species

**Figure 8:**
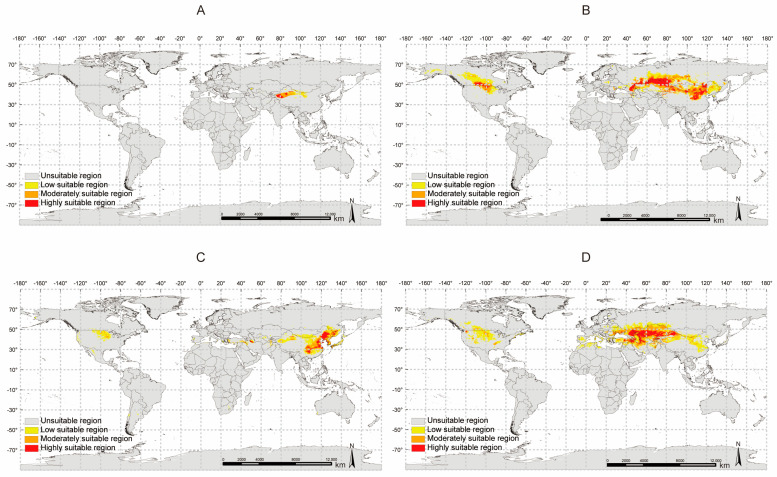
Current potentially suitable areas for four licorice species. (**A**) *Glycyrrhiza inflata*. (**B**) *Glycyrrhiza uralensis*. (**C**) *Glycyrrhiza pallidiflora*. (**D**) *Glycyrrhiza aspera*.

We collected distribution and environmental data for four *Glycyrrhiza* species and used the MaxEnt model to predict their potential distribution under current climatic conditions. The AUC values for the training and testing sets of the MaxEnt models for all four *Glycyrrhiza* species reached 0.900 (Supplementary Figure S5), indicating high reliability. Based on the MaxEnt model results, we used ArcGIS 10.8 software to map the predicted potential distribution for the four *Glycyrrhiza* species under current climatic conditions (Figure 8).

*Glycyrrhiza inflata* (Ginf), traditionally used in Chinese medicine as documented in the “Chinese Pharmacopoeia,” has a relatively narrow potential distribution under current climatic conditions, mainly concentrated in Central Asia (Figure 8A). Among the eight environmental variables involved in the distribution modeling, the Mean Diurnal Range (bio2) had the most significant impact on Ginf distribution, with a contribution rate of 19.9%. Additionally, Temperature Seasonality (bio4) and Annual Precipitation (bio12) also had high contribution rates (19.1% and 18.5%, respectively) (Supplementary Table S5).

*Glycyrrhiza uralensis* (Gura), also traditionally used in Chinese medicine, has a much broader potential distribution under current climatic conditions compared to Ginf. Its highly suitable areas are also concentrated in Central Asia, but Gura also has potential distribution in East Asia and Central North America (Figure 8B). Among the ten environmental variables involved in the distribution modeling, the Mean Temperature of Wettest Quarter (bio8) had the most significant impact on Gura distribution, with a contribution rate of 26.4%, followed by Temperature Seasonality (bio4) with a contribution rate of 22.5% (Supplementary Table S5).

*Glycyrrhiza pallidiflora* (Gpal) has a significantly different potential distribution under current climatic conditions compared to the other *Glycyrrhiza* species. It is primarily distributed in East Asia, particularly in Northeast China, North China, and the Russian Far East (Figure 8C). Among the seven environmental variables involved in the distribution modeling, Annual Mean Temperature (bio1) had the most significant impact on Gpal distribution, with a contribution rate of 28.4%. Other environmental factors with contribution rates exceeding 20% included the Precipitation of the Wettest Month (bio13) and the Max Temperature of the Warmest Month (bio5), with contribution rates of 24.4% and 22%, respectively (Supplementary Table S5).

Finally, *Glycyrrhiza aspera* (Gasp) has a different potential distribution under current climatic conditions compared to the other *Glycyrrhiza* species. Most of its suitable habitats are widely distributed across Central Asia, extending towards Eastern Europe (Figure 8D). Among the ten environmental variables involved in the distribution modeling, Temperature Seasonality (bio4) had the most significant impact on Gasp distribution, with a contribution rate of 24.4%. Other environmental factors with contribution rates exceeding 20% included the Max Temperature of the Warmest Month (bio5) and the Annual Mean Temperature (bio1), with contribution rates of 22.6% and 21.3%, respectively (Supplementary Table S5).

Overall, Annual Mean Temperature (bio1) emerged as the most critical climatic factor influencing the distribution of the four *Glycyrrhiza* species, with contribution rates exceeding 16% in the potential distribution predictions for all four species and high permutation importance values (72.6%, 16.6%, 50.4%, 59.6%). Additionally, Temperature Seasonality (bio4) is another crucial climatic factor, with contribution rates exceeding 19% in the potential distribution predictions for three *Glycyrrhiza* species (Gpal excluded bio4 from its distribution modeling to reduce the risk of overfitting). In contrast, precipitation-related factors (bio12, bio13, bio14, bio15) showed less significance compared to temperature-related factors, with only Precipitation of Wettest Month (bio13) achieving a 24.4% contribution rate in the Gpal prediction, but its permutation importance value was only 4.1%. This indicates that the potential distribution of these *Glycyrrhiza* species is primarily influenced by temperature-related climatic factors such as Annual Mean Temperature and Temperature Seasonality.

It is worth noting that although the distribution of *Glycyrrhiza* species is predominantly in Eurasia, with the highest concentration in Central Asia, we found that, except for Ginf, the other three *Glycyrrhiza* species have varying degrees of potential distribution in North America. This suggests that North America may possess ecologically suitable conditions for the growth of multiple *Glycyrrhiza* species. Through rational potential distribution prediction and planning, economic losses and resource waste caused by blind introduction can be avoided. Therefore, future efforts should focus on the effective conservation and utilization of different *Glycyrrhiza* species based on their biological and ecological characteristics under changing climatic conditions.

## 3. Materials and Methods

### 3.1. Plant Materials

Four *Glycyrrhiza* species, namely *Glycyrrhiza inflata* (Ginf), *Glycyrrhiza uralensis* (Gura), *Glycyrrhiza pallidiflora* (Gpal), and *Glycyrrhiza aspera* (Gasp), were cultivated under standard fertilization and irrigation management in the same area of desert land at the Turpan Botanical Garden, located in the southeastern part of the Turpan Basin, Xinjiang (89°11′ E, 40°51′ N). Root tissues from each of the four *Glycyrrhiza* species were harvested, cleaned of soil, washed, and dried. The samples were immediately frozen in liquid nitrogen and stored at −80 °C. After collection, the samples were stored at −80 °C for one week before metabolite estimation was conducted. This short storage period ensured that the samples remained sufficiently fresh, minimizing any potential degradation of metabolites. All samples were collected on the same day and at the same time (31 July 2023, between 4 and 5 p.m.). During sampling, the environmental temperature was 39 °C, with clear weather and no rainfall. For each *Glycyrrhiza* group, three biological replicates were used, with each biological replicate consisting of no fewer than three individual plants. This design was chosen to capture natural biological variability while ensuring the robustness of the results. The sampling procedure was conducted randomly within each species to minimize bias and ensure that the samples are representative of the natural variability in the field.

### 3.2. Metabolomics Analysis

#### 3.2.1. Sample Extraction

The metabolomic profile was generated using methods widely employed in multiple studies [65,66,67]. For LC-MS analysis, the root tissues of four types of *Glycyrrhiza* were vacuum freeze-dried using a lyophilizer (Scientz-100F, Ningbo Scientz Biotechnology Co., Ltd., Ningbo, China), then ground into powder using a grinder (MM 400, Retsch, Haan, Germany) at 30 Hz for 1.5 min. A 50 mg portion of the powdered sample was weighed, and 1200 μL of pre-cooled (−20 °C) 70% methanol–water internal standard extraction solution was added. The mixture was vortexed every 30 min for 30 s each time, for a total of six times. After centrifugation at 12,000 rpm for 3 min, the supernatant was collected, filtered through a microporous filter membrane (0.22 μm pore size), and stored in injection vials for UPLC-MS/MS analysis. Additionally, quality control (QC) samples were made by blending all sample extracts to monitor measurement repeatability.

For GC-MS analysis, the samples were ground in liquid nitrogen and vortexed thoroughly. Approximately 500 mg of each sample was weighed into headspace bottles, and saturated NaCl solution along with 20 μL of 10 μg/mL internal standard solution was added. Fully automated headspace solid-phase microextraction (HS-SPME) was employed for sample extraction, preparing the samples for subsequent GC-MS analysis. Additionally, quality control (QC) samples were made by blending all sample extracts to monitor measurement repeatability.

#### 3.2.2. Metabolite Collection and Qualitative and Quantitative Analysis

For the LC-MS platform, the sample extracts were analyzed using a UPLC-ESI-MS/MS system (UPLC, ExionLC™ AD, https://sciex.com.cn/, accessed on 15 October 2023) and a Tandem mass spectrometry system (https://sciex.com.cn/, accessed on 15 October 2023). Metabolite identification was based on a self-built MWDB (Metware Database) using secondary mass spectrometry information. Identification excluded isotopic signals, repeated signals containing K^+^, Na^+^, NH4^+^ ions, and fragment ion signals from larger molecules. Quantification was performed using the multiple reaction monitoring (MRM) mode of triple quadrupole mass spectrometry. In MRM mode, precursor ions of target substances were selected and fragmented in the collision chamber to produce fragment ions, and a specific fragment ion was selected for quantification, reducing interference from non-target ions.

For the GC-MS platform, after sampling, desorption of the VOCs from the fiber coating was carried out in the injection port of the GC apparatus (Model 8890; Agilent, Beijing, China) at 250 °C for 5 min in splitless mode. The identification and quantification of VOCs were carried out using an Agilent Model 8890 GC and a 7000E mass spectrometer (Agilent) equipped with a 30 m × 0.25 mm × 0.25 μm DB-5MS (5% phenyl-polymethylsiloxane) capillary column. Helium was used as the carrier gas at a linear velocity of 1.2 mL/min. The injector temperature was maintained at 250 °C. The oven temperature was programmed from 40 °C (3.5 min), increasing at 10 °C/min to 100 °C, at 7 °C/min to 180 °C, then at 25 °C/min to 280 °C, holding for 5 min. Mass spectra were recorded in electron impact (EI) ionization mode at 70 eV. The quadrupole mass detector, ion source, and transfer line temperatures were set at 150 °C, 230 °C, and 280 °C, respectively. The MS was operated in selected ion monitoring (SIM) mode for the identification and quantification of analytes.

### 3.3. Network Pharmacology

#### 3.3.1. Prediction of the Potential Active Ingredient

All metabolites identified in UPLC-MS/MS metabolomics were subjected to analysis using the TCMSP (https://old.tcmsp-e.com/tcmsp.php, accessed on 15 October 2023), SwissDrugDesign, and SymMap (https://www.symmap.org/, accessed on 15 October 2023) [68,69,70]. Oral utilization (OB) and drug-likeness (DL) analyses were conducted for the identified substances utilizing TCMSP. A threshold of OB > 30 and DL > 0.18 was applied to screen for active substances and to acquire information regarding the corresponding targets of these substances. Then, compound–target relationships were obtained, and the target names were converted to generic UniProt gene symbols. Simultaneously, detected substances were also screened for pharmacokinetics and drug-likeness using SWISSADME (http://www.swissadme.ch/, accessed on 15 October 2023) [71] in SwissDrugDesign, and the targets of the retained components were predicted using SwissTargetPrediction (http://www.swisstargetprediction.ch/, accessed on 15 October 2023). In SWISSADME, the pharmacokinetics analysis focused on gastrointestinal absorption (GI absorption) information, while the drug-likeness analysis considered Lipinski, Ghose, Veber, Egan, and Muegge parameters. Substances were screened under conditions where GI absorption was classified as high, and two or more of the Lipinski, Ghose, Veber, Egan, and Muegge parameters were met, while subsequent component-target analysis only retained the targets with probability values larger than 0.12. Moreover, the detected substances were also analyzed using the SymMap (https://www.symmap.org/) database to obtain corresponding target information. Finally, the corresponding target information for all active substances was collected and summarized, and The Human Disease Ontology (DO) (https://disease-ontology.org/, accessed on 15 October 2023) [72] enrichment analysis was conducted.

#### 3.3.2. Prediction of Diseases and Core Targets

Through The Human Disease Ontology (DO) enrichment analysis, the most representative disease categories were identified and used as keywords to search for disease-related genes in the DisGeNET (Version 24.3), SymMap (Version 2.0), and TTD (TTD 2024) databases. After collecting all target genes, duplicate targets were removed and the target names were standardized. Venn diagram analysis was used to identify common targets between Glycyrrhiza’s disease targets and active ingredient targets. These common targets were added to the STRING 12.0 database (https://cn.string-db.org/, accessed on 15 October 2023) [73]. Subsequently, Cytoscape 3.10.1 [74] software was used to screen for core targets, selecting those with degree, closeness, and betweenness values greater than the average as core targets.

#### 3.3.3. Gene Ontology (GO) and Kyoto Encyclopedia of Genes and Genomes (KEGG) Analysis

To elucidate the functions and metabolic pathways of the core genes, we imported them into the DAVID database (https://david.ncifcrf.gov/, accessed on 15 October 2023) [75] for Gene Ontology (GO) analysis encompassing biological process (BP), cellular component (CC), and molecular function (MF), as well as KEGG pathway analysis. Subsequently, the results of the GO and KEGG analyses were visualized and further examined through bioinformatics websites (https://www.bioinformatics.com.cn/, accessed on 15 October 2023) and (https://cloud.metware.cn/, accessed on 15 October 2023).

### 3.4. Transcriptome Analysis

According to the previous descriptions used in multiple studies [76,77,78], transcriptome analysis was conducted. RNA extraction, RNA detection, library construction, and on-machine sequencing were conducted at Metware Biotechnology Co., Ltd. (Wuhan, China). The cDNA libraries were constructed for paired-end sequencing and sequenced on the Illumina platform. Subsequently, raw sequencing data generated on the Illumina platform were filtered using fastp [79] to obtain Clean Data. The filtering criteria were as follows: paired reads were removed under the following conditions: when the number of N in any sequencing read exceeded 10% of the length of that read and when any sequencing read contained low-quality bases (Q ≤ 20) exceeding 50% of the length of that read. Subsequent analyses were based on clean reads.

After obtaining Clean Data, the reads were aligned to the reference genome of *Glycyrrhiza uralensis* [80] using HISAT2 (Version 2.2.1) [81] to obtain Mapped Data. Novel transcripts were predicted using StringTie (Version 2.1.6) [82], and the predicted novel genes were annotated using diamond [83] against databases including KEGG, GO, NR, Swiss-Prot, TrEMBL, and KOG with an E-value threshold of 1 × 10^−5^. Plant transcription factors were predicted using Itak (Version 1.6) [84] software. Gene quantification was performed using featureCounts (Version 2.0.3) [85], and FPKM values were calculated to estimate gene expression levels. Differential expression analysis between the two groups was conducted using DESeq2 (Version 1.22.1) [86,87], with *p*-values adjusted using the Benjamini–Hochberg method. Genes were considered differentially expressed if |log_2_Fold Change| ≥ 1 and FDR < 0.05. Enrichment analysis based on hypergeometric testing was performed for KEGG pathways and GO terms.

### 3.5. Potential Distribution Prediction

#### 3.5.1. Species Distribution and Environmental Data Collection and Processing

Species distribution data were sourced from the Global Biodiversity Information Facility (GBIF, http://www.gbif.org, accessed on 15 October 2023). Locations with precise latitude and longitude records were cross-checked to remove outlier distribution points and exclude cultivated or introduced locations, thereby organizing distribution information. Environmental data under current climate scenarios were downloaded from Worldclim (http://www.worldclim.org/, accessed on 15 October 2023) [88], comprising 19 environmental variables (bio1~bio19) at a spatial resolution of 2.5 min. To mitigate the risk of overfitting models due to collinearity among environmental factors [89] and to enhance prediction accuracy, we initially filtered out correlated environmental variables and conducted preliminary modeling experiments for the four licorice species. Subsequently, using ArcGIS 10.8, we performed numerical sampling of licorice distribution points and environmental factors, calculating Pearson correlation coefficients between genes/metabolites using the COR program in R 4.3.2. Finally, based on the results of preliminary modeling experiments and correlation analysis, climate factors with |r| < 0.8 were retained for further bioclimatic suitability zone prediction.

#### 3.5.2. Bioclimatic Suitability Zone Prediction Based on MaxEnt Model

Based on the selected environmental variables, the MaxEnt 3.4.4 model, in conjunction with ArcGIS 10.8, was used to predict the potential distribution of licorice under different climatic scenarios. The parameters for MaxEnt were configured as follows: the training dataset was set to 75%, while the testing validation dataset was set to 25%. The model underwent 10,000 iterations with 20 repetitions for robustness. The Jackknife test was employed to assess the contribution of each bioclimatic variable. Model accuracy was evaluated using Receiver Operating Characteristic (ROC) curves and calculating the Area Under the Curve (AUC) values. AUC values from the ROC curves of the training and testing datasets were used as indicators of model accuracy. Default settings of MaxEnt 3.4.4 software were used for other parameters.

The results from the MaxEnt model simulations were imported into ArcGIS 10.8 for reclassification and visualization. Using the Jenks’ natural breaks classification method, the potential distribution of the four licorice species was classified into four categories: unsuitable region, low suitable region, moderately suitable region, and highly suitable region.

## 4. Conclusions

This study utilized UPLC-MS/MS and GC-MS-based metabolomics to conduct a comparative analysis of secondary metabolites in four *Glycyrrhiza* species (Ginf, Gura, Gpal, Gasp). A total of 2716 metabolites were identified. Using network pharmacology, 299 key active components of traditional Chinese medicine were selected for Disease Ontology (DO) enrichment analysis, revealing significant enrichment in diseases related to the cardiovascular system. Subsequently, we predicted the potential pharmacological components and mechanisms of action of the four *Glycyrrhiza* species against cardiovascular system diseases.

Furthermore, we employed an integrative approach combining transcriptomics and metabolomics to study the differentially expressed genes (DEGs) and secondary metabolite accumulation in the four *Glycyrrhiza* species. KEGG annotation and enrichment analysis were performed on DEGs and differentially expressed metabolites (DEMs) in the comparisons Ginf vs. Gpal, Gura vs. Gpal, Gura vs. Gasp, and Ginf vs. Gasp. The results indicated significant differences in the Isoflavonoid biosynthesis pathway among the *Glycyrrhiza* species. From this, we identified five metabolites and 10 genes as candidate hub genes and metabolites related to isoflavonoid biosynthesis in different *Glycyrrhiza* species.

Finally, by integrating distribution and environmental data, the MaxEnt model was used to predict the potential distribution of the four *Glycyrrhiza* species under current climatic conditions, providing a practical basis for further development and utilization of *Glycyrrhiza* resources.

This study provides comprehensive information on the metabolites in *Glycyrrhiza* species that have health-promoting functions, preliminarily elucidates the potential pharmacological components and mechanisms for treating cardiovascular system diseases, and reveals the biosynthesis mechanisms of isoflavonoid compounds in different *Glycyrrhiza* species through combined transcriptomic and metabolomic analysis. Additionally, it predicts the potential distribution of the four *Glycyrrhiza* species under current climatic conditions, aiding in the better and more comprehensive development and utilization of *Glycyrrhiza* resources.

## Figures and Tables

**Table 1:** Statistical analysis of reads mapping to the reference genome in each group.

Sample	Total Reads	Reads Mapped
Gasp_1	47,498,206	38,202,345 (80.43%)
Gasp_2	44,688,516	35,707,659 (79.90%)
Gasp_3	46,353,134	37,110,877 (80.06%)
Ginf_1	43,803,934	40,780,463 (93.10%)
Ginf_2	53,088,030	49,443,635 (93.14%)
Ginf_3	43,954,566	40,963,011 (93.19%)
Gpal_1	48,659,588	44,585,380 (91.63%)
Gpal_2	45,988,796	42,218,272 (91.80%)
Gpal_3	42,524,312	39,088,852 (91.92%)
Gura_1	46,388,868	44,009,841 (94.87%)
Gura_2	44,436,814	42,180,943 (94.92%)
Gura_3	45,695,936	43,363,295 (94.90%)

## Data Availability

Data are contained within the article and Supplementary Materials.

## Supplementary Materials

The supporting information can be downloaded at https://www.mdpi.com/article/10.3390/ijms26062539/s1.
