# Supplementary figures and images for: Integrated Metabolomic and Transcriptomic Analysis Reveals the Pharmacological Effects and Differential Mechanisms of Isoflavone Biosynthesis in Four Species of Glycyrrhiza
Source: Int J Mol Sci. 2025 Mar 12;26(6):2539. doi: 10.3390/ijms26062539 (PMC11942288; doi:10.3390/ijms26062539)

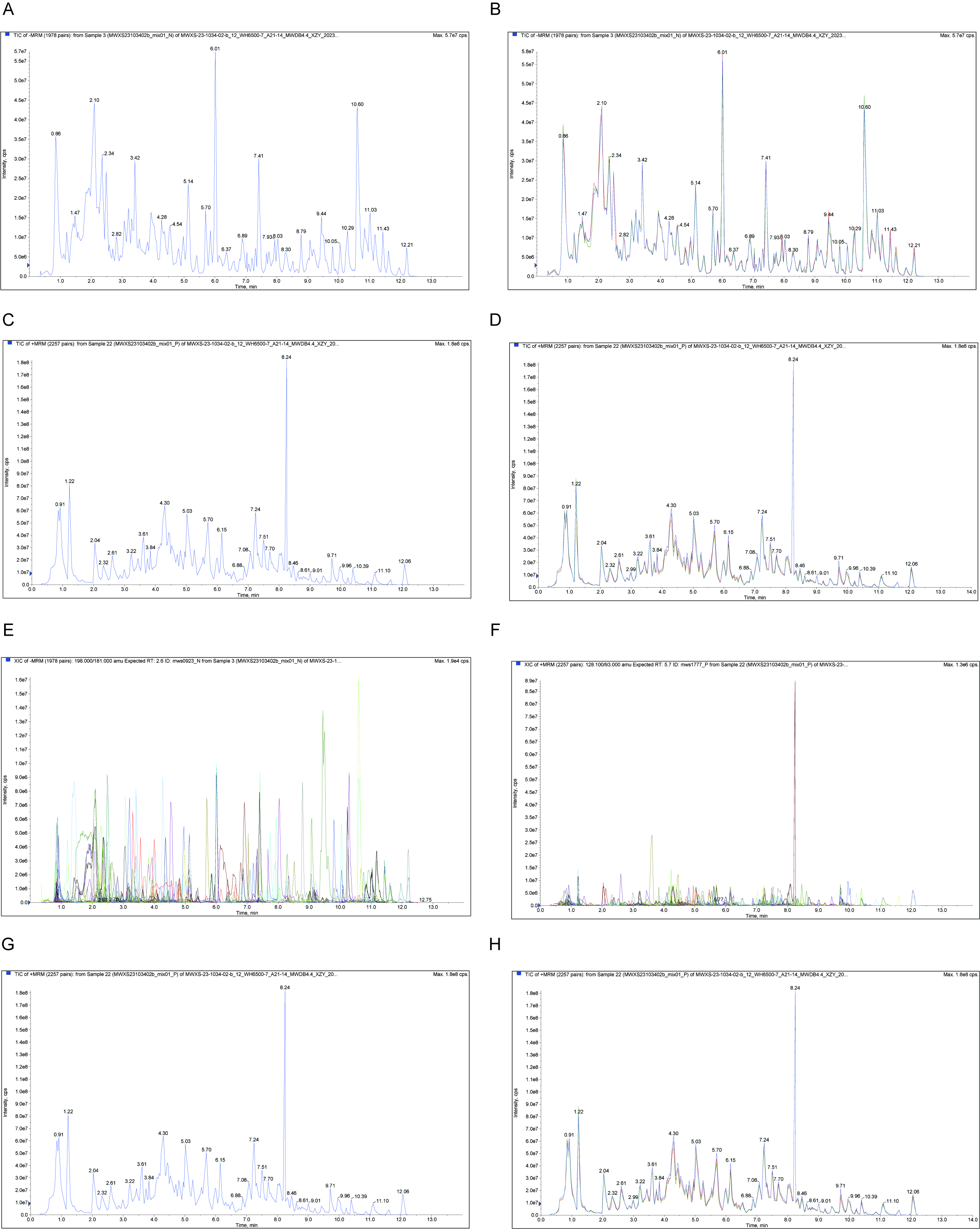

Supplement: Supplementary file 1 [file ijms-26-02539-s001.zip › figs1.tif]

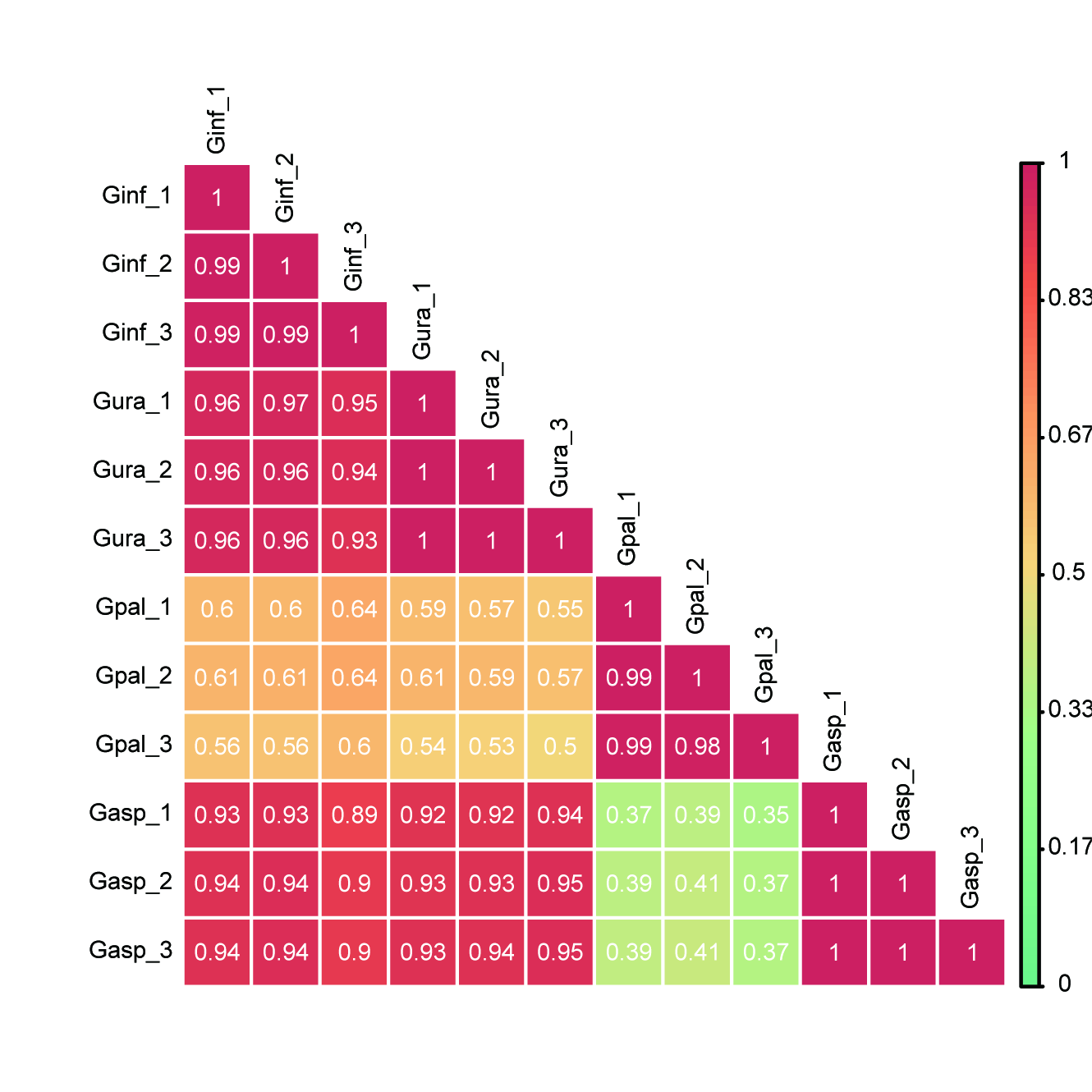

Supplement: Supplementary file 1 [file ijms-26-02539-s001.zip › figs2.tif]

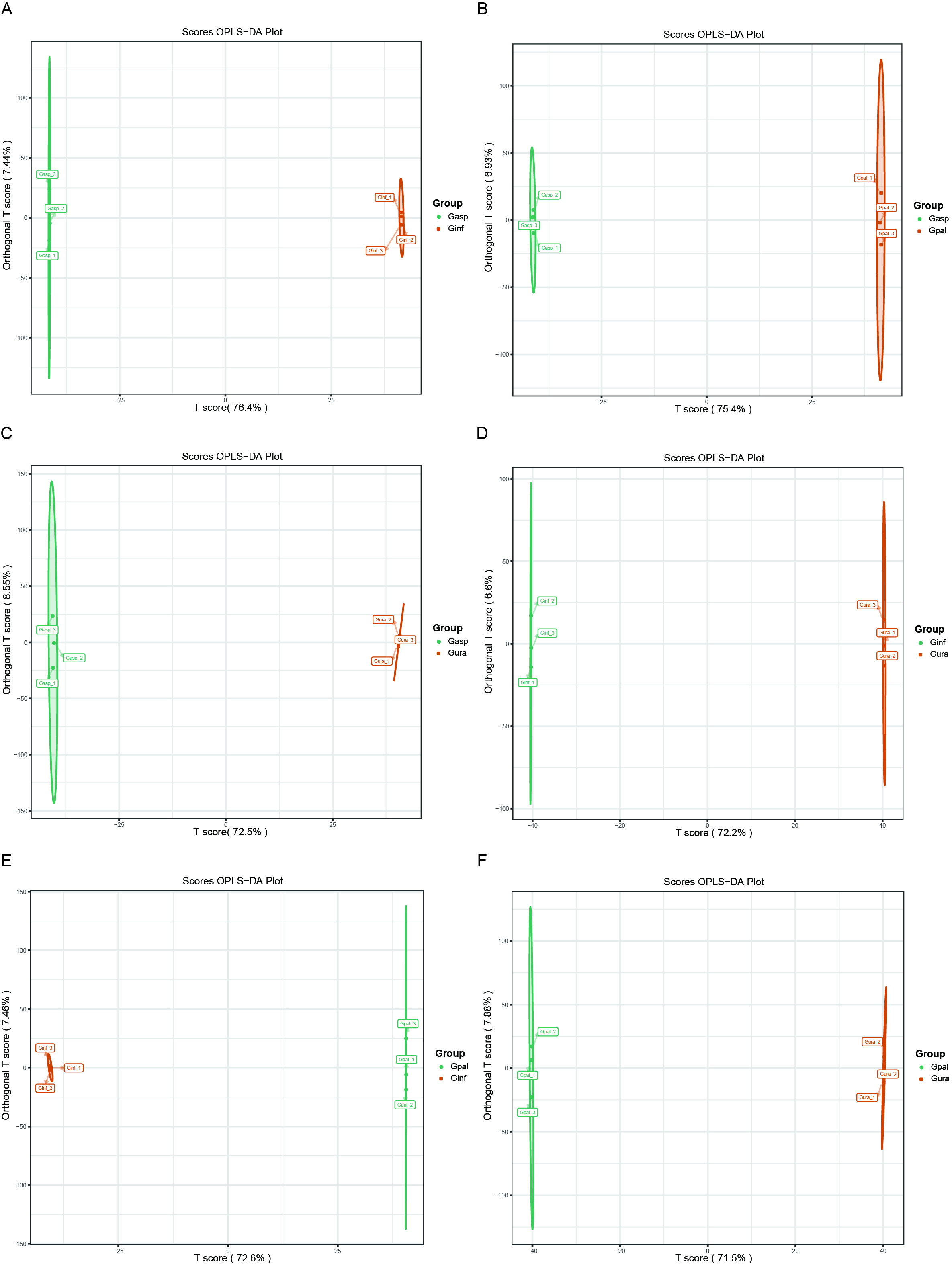

Supplement: Supplementary file 1 [file ijms-26-02539-s001.zip › figs3.tif]

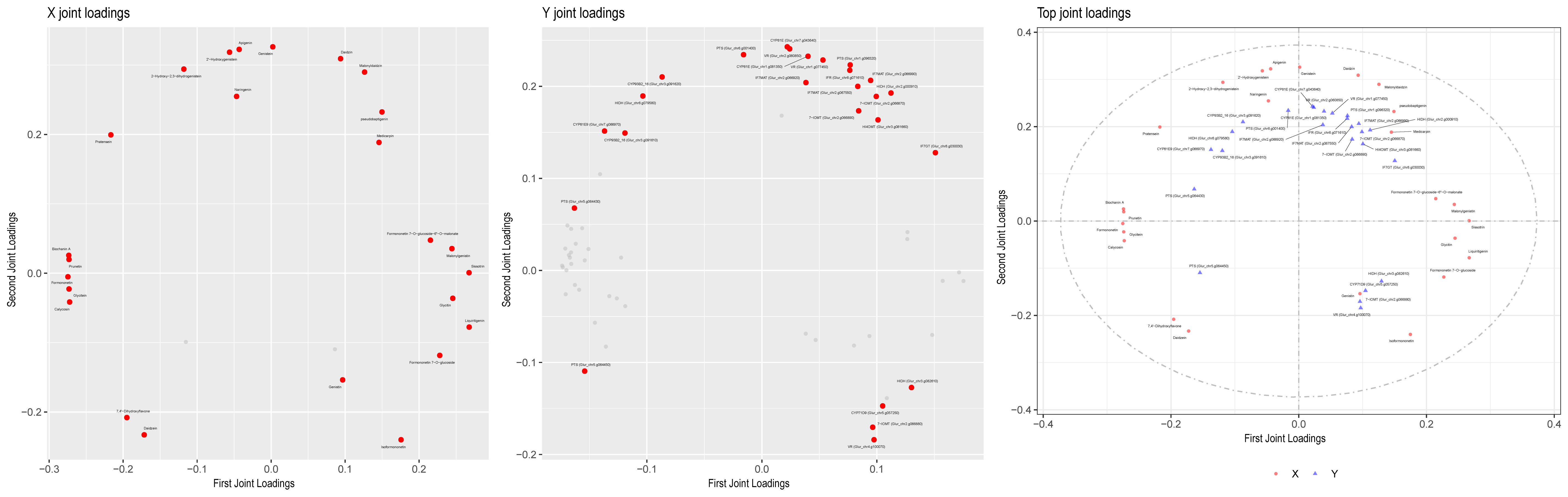

Supplement: Supplementary file 1 [file ijms-26-02539-s001.zip › figs4.tif]

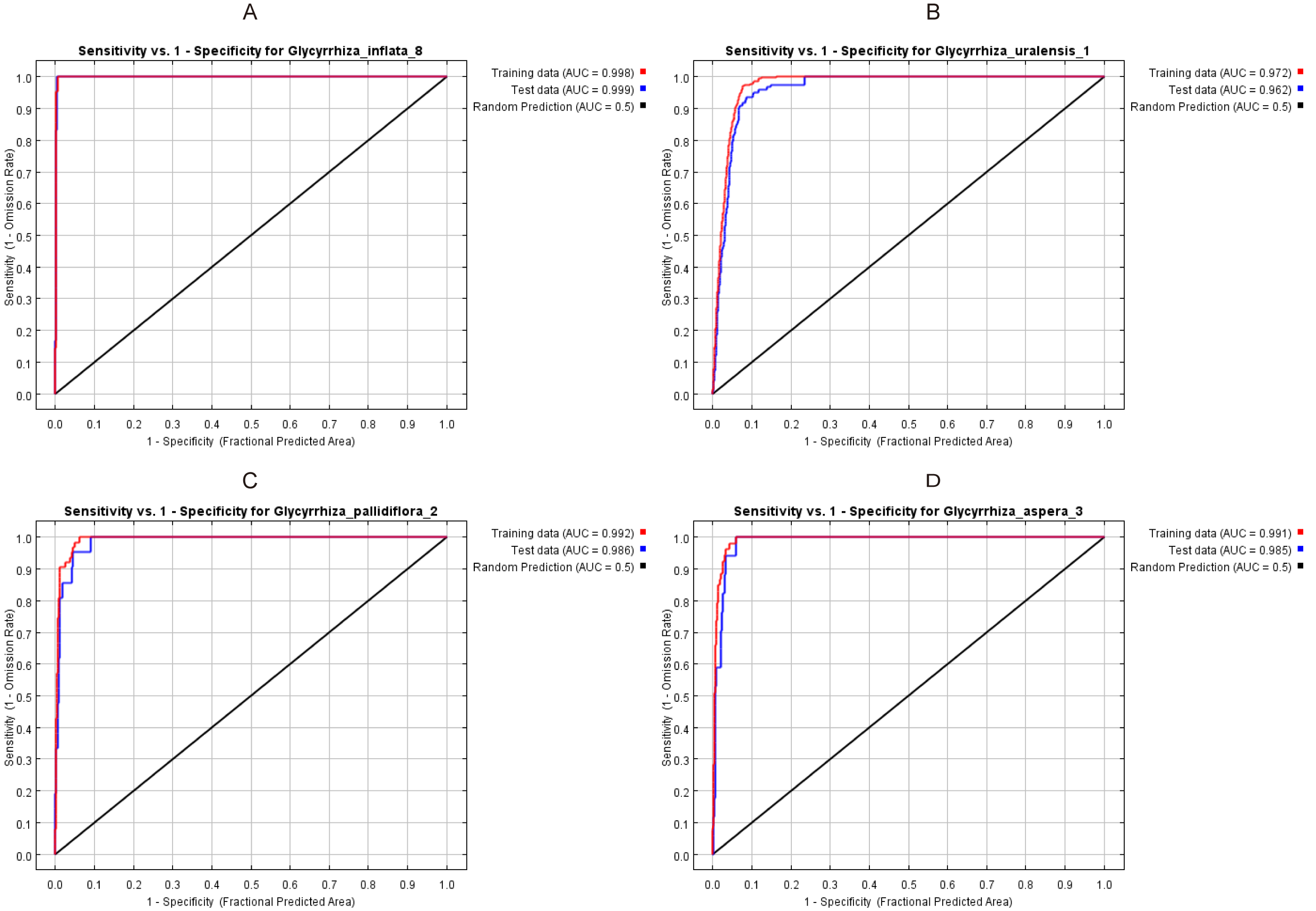

Supplement: Supplementary file 1 [file ijms-26-02539-s001.zip › figs5.tif]
